# Generation of xenobiotic free retinofugal assembloids

**DOI:** 10.3389/fcell.2025.1746709

**Published:** 2026-01-14

**Authors:** Michael H. Hayes, Maria F. Valdes Michel, Markus H. Kuehn, Randy H. Kardon, Oliver W. Gramlich

**Affiliations:** 1 Center for the Prevention and Treatment of Visual Loss, US Department of Veterans Affairs, Iowa City, IA, United States; 2 Department of Ophthalmology and Visual Sciences, University of Iowa, Iowa City, IA, United States; 3 Department of Ophthalmology and Visual Sciences, University of Alabama at Birmingham, Birmingham, AL, United States

**Keywords:** assembloid, cortical organoid, new approach method, optic neuritis, retinal ganglion cell, retinal organoid, xenobiotic free, xeno-free

## Abstract

Retinal ganglion cells (RGCs) are the output neurons of the retina, responsible for transmitting visual signals to the brain through the optic nerve. Their long axons, high metabolic demand, and the variable environments they transit make them particularly vulnerable to neurodegenerative insults in optic neuropathies. These insults include oxidative stress, excitotoxicity, and inflammatory damage, either within the neuroretina or within the optic nerve, and are thought to drive disease etiology. RGC-related vision loss is the primary presenting concern in many optic neuropathies including glaucoma and autoimmune demyelinating diseases such as multiple sclerosis MS-related optic neuritis (ON) is a result of immune-mediated damage to the myelinated optic nerve, a process not fully recapitulated in current *in vitro* organoid models. For instance, 3D organoid models offer improved architectural context, but they lack crucial cell types and sufficient anatomic complexity to mimic the *in vivo* environment. Further, widespread use of animal-derived reagents in these systems can introduce significant phenotype variability posing a major barrier to translational research. To address these challenges, retinofugal assembloid models have emerged. These models combine retinal and brain organoids to recapitulate the *in vivo* visual pathway, supporting RGC survival, RGC axonal extension and pathfinding, incorporation of additional glial cell types, and provide sufficient complexity. Here, we describe xenobiotic-free protocols for generating retinal and oligodendrocyte-rich cortical organoids and their fusion into assembloids to more accurately model RGC physiology. We discuss the advantages, limitations, and future applications of these systems in studying neuroinflammation and demyelination in a human-relevant context.

## 1 Introduction

Retinal ganglion cells (RGCs) are the sole output neurons of the retina. There is significant diversity in these cells, with a minority that are intrinsically photosensitive, participating in non-vision forming behaviors such as the pupillary response, mood modulation, and maintenance of circadian rhythm (Kim et al., 2021). The majority of RGCs project visual information from the retina to vision processing centers in the brain starting with the thalamic lateral geniculate nucleus. These long-range axonal projections coalesce as they exit the eye to form the optic nerve (Dhande et al., 2015). Beyond serving as the primary conduit for visual information, RGCs play a critical role in orchestrating the structural and functional development of the mammalian visual system, including the establishment of retinotopic maps and synaptic connectivity (D'Souza and Lang, 2020; Zhang and Johnston, 2023).

Besides their essential role in visual processing and development, RGCs are often targets in neurodegenerative diseases due to their heightened vulnerability. Their long axons require substantial energy to maintain axonal transport and synaptic activity, making them particularly sensitive to mitochondrial dysfunction and oxidative stress (Osborne et al., 2004). RGCs are also susceptible to glutamate-induced excitotoxicity and inflammatory cytokines, which are elevated in various neurodegenerative conditions, including optic neuropathies (Tezel, 2006). Optic neuropathies encompass a diverse group of disorders characterized by optic nerve degeneration and visual impairment. These include hereditary conditions such as Leber hereditary optic neuropathy and dominant optic atrophy, as well as ischemic optic neuropathy and toxic neuropathies induced by drugs or environmental conditions (De Lott, 2025; Biousse and Newman, 2016). Optic neuropathies also include glaucoma, a leading cause of irreversible blindness worldwide, (Stein et al., 2021), as well as several inflammatory autoimmune demyelinating diseases such as multiple sclerosis (MS), neuromyelitis optica spectrum disorder (NMOSD) and myelin oligodendrocyte glycoprotein antibody disease (MOGAD) (Ciapă et al., 2022; Chaitanuwong and Moss, 2025). Optic neuritis (ON), a common initial manifestation of these conditions, reflects the optic nerve’s unique vulnerability arising from its myelination, immune exposure, high metabolic demand, and transitional axonal architecture (Moheb and Chen, 2023; Lee et al., 2017).

Despite advances in understanding disease mechanisms, modeling these processes in human-relevant systems remains a challenge. Animal models have been instrumental in uncovering pathobiology and guiding therapeutic development, but they fail to fully replicate human disease complexity (Capper et al., 2025) and translation to successful treatment in humans. To address this gap, researchers have increasingly adopted human *in vitro* systems (Godwin et al., 2022). These models, using human-derived tissue in 2D and 3D formats, enable controlled exploration of human-specific biology. 2D RGC monocultures offer accessibility, standardization, and imaging compatibility, and have significantly advanced our understanding of RGC biology (Whitehead et al., 2025). However, monocultures lack the cellular diversity of the *in vivo* environment. To overcome this, 2D co-culture models incorporating glial cells such as astrocytes and microglia have been developed, allowing investigation of RGC-glia interactions, cell-cell signaling, and drug responses (Liu et al., 2022; Lish et al., 2025; Harkin et al., 2025). These systems support high-throughput screening and transcriptomic analyses but still fall short of replicating the three-dimensional microenvironment, molecular gradients, and structural organization that shape cellular behavior *in vivo* (Huh et al., 2011).

3D culture models offer improved spatial organization and multicellular architecture, better mimicking human systems. However, most rely on single-tissue organoids, which lack key cell types, neighboring tissues, and intercellular cues necessary for modeling complex biology and disease. Modeling MS-associated ON highlights these limitations. ON, presenting in ∼70% of MS cases (Ciapă et al., 2022), results from autoimmune inflammation and subsequent demyelination of the optic nerve, a process requiring oligodendrocytes, which are absent in retinal organoids (Kale, 2016; Huang et al., 2024; Harkin et al., 2024). Integrating RGCs with oligodendrocytes from brain-derived organoids is therefore essential to replicate MS-related ON.

**FIGURE 1:**
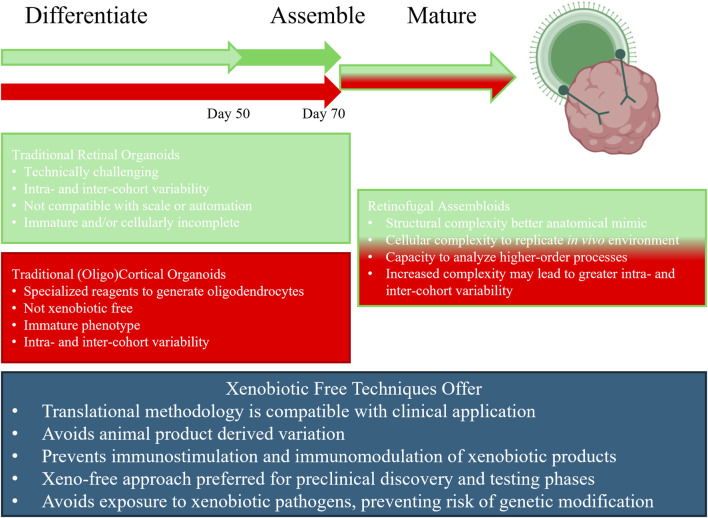
Retinofugal assembloids and xenobiotic free techniques offer multiple advantages. Differentiation of retinal (green) and cortical (red) (Nieto-Estévez et al.) organoids is a 50-day process. Induction and maturation of oligodendrocytes is a 20-day process making oligocortical organoid differentiation a 70-day protocol. Single tissue organoids lack the complexity required to model complicated disease processes. Additionally, traditional methods rely on animal derived products that can increase variability, induce an immune response, and expose cultures to xenobiotic pathogens that can genetically modify cultured tissues and influence experimental outcomes. Xenobiotic free techniques overcome these pitfalls while maintaining translatability.

To address these limitations, we and others have developed assembloid models that integrate retinal organoids with other brain-derived structures (Figure 1) (Hayes et al., 2025; Salti et al., 2024; Fligor et al., 2021). Fligor et al. demonstrated that induced pluripotent stem cell (iPSC)-derived retinal organoids fused with cortical and thalamic organoids can support RGC axon extension and survival, modeling retinofugal projections (Fligor et al., 2021). The Bolz lab developed stem-cell-derived retinal and cortical organoid assembloids to model early RGC differentiation, axon growth, and pathfinding, as well as their disruption in inherited retinal dystrophy (Salti et al., 2024). Across these studies, coalescence of retina and brain region organoids into retinofugal assembloids results in enhanced RGC survival and axon pathfinding into the brain-like tissue (Fligor et al., 2021). To further advance this pioneering work, we describe step-by step, xenobiotic-free protocols to differentiate human-derived iPSCs into retinal and oligocortical organoids using the reagent detailed in Table 1. These human organoids are subsequently combined to form higher-order retinofugal assembloids that accurately model RGC development, differentiation, and maturation within a xenobiotic-free microenvironment to increase the scientific rigor of this highly innovative and translational research platform.

**TABLE 1:** Oligocortical and retinal organoid differentiation reagents and supplements.

Reagent	Company	Catalog number	Function
CTS TrypLE select enzyme	Gibco	A1285901	Cell dissociation
DPBS	Gibco	14-190-144	Buffer for washing cells
CTS KnockOut serum replacement	Gibco	12618013	Serum replacement for differentiation
MEM non-essential amino acids	Gibco	11140050	Supplement for cell culture
GlutaMAX	Gibco	35050061	Glutamine supplement
β-mercaptoethanol	Sigma	M3148	Antioxidant supplement
Primocin	InvivoGen	ant-pm-1	Antibiotic for contamination prevention
ROCK inhibitor Y-27632	Tocris	1254	Enhances cell survival during dissociation
Dorsomorphin	Sigma	P5499	Neural induction supplement
SB-431542	Tocris	1614	Neural induction supplement
Neurobasal-A	Thermo Fisher	10888022	Base medium for neuronal differentiation
B-27 supplement	Gibco	17504044	Neuronal supplement
FGF-2	R&D systems	233-FB	Growth factor for neuronal differentiation
EGF	R&D systems	236-EG	Growth factor for neuronal differentiation
BDNF	R&D systems	248-BD	Neurotrophic factor for neuronal maturation
NT-3	R&D systems	268-N3	Neurotrophic factor for neuronal and glial development
PDGF-AA	R&D systems	221-AA	Promotes OPC proliferation and maturation
IGF-1	R&D systems	291-G1	Supports neuronal and glial survival and myelination
T3 (Triiodothyronine)	Sigma	T6397	Thyroid hormone for oligodendrocyte maturation
CTS KnockOut DMEM	Gibco	A1286101	Base medium for retinal differentiation
CTS KnockOut DMEM/F12	Gibco	A1370801	Base medium for SSM and NR media
CTS N-2 supplement	Gibco	A1370701	Supplement for NR medium
Sodium pyruvate	Gibco	11360-070	Energy source supplement
Human serum (shestopalov et al.)	Innovative research	IPLA-SERAB-HI	Supplement for 3D differentiation medium

## 2 Materials and equipment

### 2.1 Cell lines

#### 2.1.1 iPSC lines

- Human healthy donor iPSC line (ACS-1026; ATCC, Manassas, VA, USA)
- Brn3-GFP reporter transgenic iPSC line as previously described (Lei et al., 2024; Godwin et al., 2022; Cheng et al., 2022).

### 2.2 Plates and equipment

- Low-adherence V-bottom 96-well plates (SBio, Cat# MS-9096VZ)
- Ultra-low attachment 6-well plates (Corning, Cat# CLS3471)
- Digital microscope
- Tabletop centrifuge
- Incubator/cell culture system
- 15 mL and 50 mL polypropylene screw-cap conical tubes
- Water bath
- CTS TrypLE Select Enzyme (Gibco, Cat# A1285901)
- DPBS (Gibco, Cat# 14-190-144)
- Cell counter
- Lab marker.

### 2.3 Media

#### 2.3.1 iPSCs medium

- Complete mTeSR Plus™ (StemCell Technologies)
- Supplemented with Primocin (InvivoGen) when contamination risk is present

#### 2.3.2 Spheroid starter medium (SSM)

- 400 mL DMEM/F-12 (Invitrogen, Cat# 411320-033)
- 100 mL CTS KnockOut Serum Replacement (Gibco, Cat# 12-618-013)
- 5 mL MEM non-essential amino acids (Gibco, Cat# 11-140-050)
- 5 mL GlutaMAX (Gibco, Cat# 35-050-061)
- 5 µL β-mercaptoethanol (from 100 µM stock; Sigma, Cat# M3148-25 ML)
- 1 mL Primocin (InvivoGen)
- Supplement 50 mL aliquots with:
o 10 μM ROCK inhibitor Y-27632 (Sigma, Cat# Y0503-1 MG)
o 5 μM Dorsomorphin (Sigma, Cat# P5499-5 MG)
o 10 µM SB-431542 (Tocris, Cat# 1614)

#### 2.3.3 Neurobasal medium (NB)

- 484 mL Neurobasal-A (Thermo Fisher, Cat# 10888022)
- 10 mL B-27 Supplement (Gibco, Cat# 17504044)
- 5 mL GlutaMAX (Gibco)
- 1 mL Primocin
o Supplement 50 mL aliquots with:
o 20 ng/mL FGF-2 (R&D Systems, Cat# 3718-FB)
o 10 ng/mL EGF (R&D Systems, Cat# 236-EG-200)
o 1% recombinant human extracellular matrix (ECM) mix
o 20 ng/mL BDNF (R&D Systems)
o 20 ng/mL NT-3 (R&D Systems)
o 10 ng/mL PDGF-AA (R&D Systems)
o 10 ng/mL IGF-1 (R&D Systems)
o 40 ng/ml T3 (Sigma, Cat# ST2877)

#### 2.3.4 3D differentiation medium

- 395 mL CTS KnockOut DMEM (Gibco, Cat# A1286101)
- 50 mL heat-inactivated human serum (Innovative Research, Cat# IPLA-SERAB-HI)
o Insoluble material removed via filtration through 0.80-, 0.45-, and 0.22-micron filters.
    ⁃ 0.80micron PES 25 mm Syringe Filter (JinTeng, Cat# PES-0.8-25)
    ⁃ 0.45micron PES Filter System (Dot Scientific, Cat#229702)
    ⁃ 0.22micron PES Filter System (Dot Scientific, Cat#229706)
- 100 mL CTS KnockOut Serum Replacement (Gibco, Cat# 12-618-013)
- 5 mL MEM non-essential amino acids (Gibco, Cat# 11-140-050)
- 5 mL CTS GlutaMAX (Gico, Cat# A1286001)
- 5 mL sodium pyruvate (Gibco, Cat# 11360-070)
- 200 µL β-mercaptoethanol (Sigma, Cat# M3148-25 ML)
- 1 mL Primocin
o Supplement 50 mL aliquots with:
o 10 μM ROCK inhibitor Y-27632 (Tocris, Cat# 1254)
o 1.5 μM (55 ng/mL) BMP4 (R&D Systems)

#### 2.3.5 Neural retina (NR) medium

- 489 mL CTS KnockOut DMEM/F12 (Gibco, Cat# A1370801)
- 5 mL CTS N-2 Supplement (Gibco, Cat# A1370701)
- 5 mL CTS GlutaMAX (Gibco)
- 1 mL Primocin

## 3 Methods

### 3.1 iPSCs culturing

#### 3.1.1 iPSCs maintenance

- Culture media (mTeSR Plus) was changed, and cultures were monitored for signs of spontaneous differentiation daily, all differentiated cells were manually removed with a p10 pipette.

#### 3.1.2 iPSCs passaging

A detailed list of iPSC culturing reagents can be found in Table 2.

- IPSCs were passaged as aggregates at a 1:6 to 1:12 ratio when they reached ∼70% confluence.
- iPSCs were passaged as aggregates to help maintain pluripotency. Spent mTeSR medium was aspirated, and cells were rinsed with 2 mL of 1× DPBS. Each well was then incubated with 1 mL of Versene (Gibco, Cat# 5040066) for 5 min at room temperature. Versene, an EDTA-based dissociation reagent, chelates calcium ions required for integrin-mediated adhesion, thereby preserving cell–cell contacts within aggregates. This approach minimizes single-cell dissociation and reduces spontaneous differentiation when passaging (Yamamoto et al., 2018; Lai et al., 2022).
- All iPSCs used for downstream differentiations were below passage 50

**TABLE 2:** iPSC culturing reagents.

Reagent	Company	Catalog number	Function
TC-treated 6-well plates	Corning	07-200-80	Cultureware for Laminin-521 coating
Non-TC 6-well plates	STEMCELL Technologies	27147	Cultureware for Vitronectin XF coating
Versene solution	Gibco/Fisher scientific	15-040-066	Cell dissociation for passaging
DPBS	Gibco	14-190-144	Buffer for washing cells
BAMBANKER	Bulldog Bio Inc/Fisher scientific	NC2960954	Cryopreservation of iPSCs
Vitronectin XF	STEMCELL Technologies	07180	Coating substrate to maintain pluripotency
CellAdhere dilution buffer	STEMCELL Technologies	07183	Dilution buffer for Vitronectin XF and Laminin-521
Laminin-521	STEMCELL Technologies	77003	Coating substrate for differentiation
mTeSR-Plus	STEMCELL Technologies	85850	Complete medium for iPSC maintenance

### 3.2 Generation of oligocortical organoids

#### 3.2.1 Differentiation protocol

##### 3.2.1.1 Day 0–2: iPSCs preparation

A detailed list of oligocortical organoid differentiation and culturing reagents can be found in Table 1.

- Warm mTeSR Plus medium in water bath (≥30 min)
- Wash each well with 2 mL of 1xDPBS
- Add 1 mL room temperature CTS TrypLE Select Enzyme (Gibco) per well to iPSC colonies that are free of differentiating cells and at ∼70% confluence
o We routinely dissociate N+1 well, where N is equal to the number of 96 well plates we intend to seed. This step creates a large surplus of cells to start multiple differentiations.
- Incubate ∼5 min at 37 °C until colony edges curl
- Add 2 mL of prewarmed mTeSR Plus to stop dissociation
- Pipette up and down to release and separate cells to create a single-cell solution
- Transfer cells to 15 mL conical tubes
- Centrifuge the cells to pellet (3 min, 200 x g, at room temperature (RT)) and aspirate supernatant
- Resuspend in 1 mL of pre-warmed mTeSR Plus +10 μM ROCK inhibitor Y-27632
- Count cells using hemocytometer (1 × 10^5^ cells/mL)
- Seed 10,000 cells in 100 µL per well in ultra-low attachment V-bottom 96-well plates (SBio, Cat# MS-9096VZ)
- Centrifuge plates (300 × g, 3 min) to pellet cells at the bottom of the well
- Incubate at 37 °C, 5% CO_2_

##### 3.2.1.2 Day 3–70: Spheroid differentiation

- Day 3: Add 100 µL SSM +5 µM Dorsomorphin +10 µM SB-431542 to each well
o It is important to note that dorsomorphin can be cytotoxic and will likely need to be empirically adjusted. We recommend testing concentrations between 1 and 10 µM
- Days 4–7: Perform daily half-media changes with fresh SSM +5 µM Dorsomorphin +10 µM SB-431542
- Days 8–9: Switch to NB + 10 ng/mL EGF +20 ng/mL FGF-2; half-media changes
- Days 11–23: Continue NB + EGF + FGF-2 + 1% ECM; half-media changes every other day such as a Monday-Wednesday-Friday (MWF) schedule
- Day 25: Transfer spheroids to ultra-low attachment 6-well plates or 10 cm dishes; switch to NB + 1% ECM (no growth factors)
- Days 28–39: NB + 20 ng/mL BDNF +20 ng/mL NT-3 + 1% ECM; full media changes every other day (MWF)
- Days 42–49: NB + 1% ECM only; full media changes (MWF)
- Days 50–58: NB + 1% ECM +10 ng/mL PDGF-AA + 10 ng/mL IGF-1; full media changes (MWF)
- Days 60–67: NB + 1% ECM +40 ng/ml T3; full media changes (MWF)
- Day 70 onward: Maintain in NB + 1% ECM; full media changes every other day (MWF)

### 3.3 Generation of retinal organoids

#### 3.3.1 Differentiation protocol

##### 3.3.1.1 Day 0–2: iPSCs preparation

A detailed list of oligocortical organoid differentiation and culturing reagents can be found in Table 1.

- Sterilize incubator and cell culture room
- Warm 3D differentiation medium (≥30 min) in bead water bath at 37 °C
- Add 1 mL room temperature TrypLE to iPSC colonies
- Incubate ∼5 min at 37 °C until colony edges curl
- Add 2 mL of prewarmed mTeSR Plus to stop dissociation
- Pipette up and down to release and separate cells to create a single-cell solution
- Collect cells into 15 mL conical tube
- Centrifuge (3 min, 200 × g, RT), aspirate, resuspend in 1 mL 3D medium
- Count cells with hematocytometer
- Seed 4 × 10^5^ cells in 10 mL 3D medium + ROCK inhibitor per 96-well plate
- Dispense 4,000 cells in 100 µL per well
- Centrifuge (300 × g, 3 min), incubate at 37 °C, 5% CO_2_

##### 3.3.1.2 Day 3–40: Retinal cup formation

- Day 3: Add 100 µL 3D medium + ROCK inhibitor +1% ECM
- Day 6: Replace 100 µL with fresh 3D medium + BMP4 + 1% ECM
- Days 8–15: Half-media changes (MWF) to gradually reduce BMP4
- Day 16: Transfer organoids to ultra-low attachment plates; add NR medium +1% ECM
- Day 18 onward: Continue NR medium +1% ECM; media changes every MWF
- Days 30–40: Identify and manually dissect neural epithelium “cups”

##### 3.3.1.3 Post-day 40 maintenance

- Warm NR medium (≥30 min) at 37 °C
- Carefully remove spent media
- Add:
o 2 mL NR to 6-well plates
o 10 mL NR to 10 cm dishes
- Incubate at 37 °C, 5% CO_2_, 20% O_2_
- Media changes every other day (MWF)

### 3.4 Establishment of assembloid cultures

Retinal and oligocortical organoids were differentiated as described above and assembled on day 70. GFP + RGC containing retinal organoids were bisected with a sterile scalpel blade and the cut surface was orientated facing a whole Di-I stained oligocortical organoid in a low-adherence 12-well plate. To facilitate assembly, one edge of the 12-well plate was placed on top of an empty plate to obtain a ∼45° tilt, allowing gravity to bring the organoids together. Prior to assembloid formation, oligocortical organoids were stained with Di-I (ThermoFisher, Cat# D282), per manufacturer instructions, to allow for easy identification of oligocortical derived cells. Assembloids were left undisturbed for 3 days before imaging. The retinofugal assembloids were established and subsequently cultured in NR media. Media changes were performed every other day on a Monday-Wednesday-Friday (MWF) schedule thereafter.

For live imaging experiments assembloids were generated from two different lines. Retinal organoids were derived from the Brn3-GFP reporter iPSC line to facilitate easy identification of RGCs via GFP expression. The naïve ATCC iPSC line was used to generate oligocortical organoids to eliminate the possibility of non-RGC GFP expression as Brn3 is expressed within multiple subsets of neurons within the CNS (Badea et al., 2012). To image, assembloids were placed into 35 mm glass bottom dishes (Mattek P35G-1.5-14-C) with culture media or 1X DPBS. To improve resolution and depth, some images were acquired following overnight with 18% v/v Iodixanol (“Visipaque” GE) in culture medium to achieve optimal matching of refractive index between the assembloid and its medium (Figures 3D, 4C–E) (Boothe et al., 2017). Phase contrast images were captured using an Olympus CK40 with a Panasonic Lumix GF3 adaptor. Fluorescence imaging was performed on an inverted Zeiss ObserverZ.1 epifluorescence microscope or inverted Nikon eclipse Ti2 confocal microscope.

### 3.5 Fixation, cryosectioning, and immunofluorescence

Whole organoids were fixed for at least 2 h in 4% paraformaldehyde solution at 4 °C then cryoprotected overnight in 1XDPBS containing 30% sucrose (RPI Research, Cat# S24060) and 0.1% sodium azide (Alfa Aesar, Cat# 14314) to prevent microbial growth. Fixed organoids were mounted in optimum cutting temperature compound (OCT, ThermoFisher, Cat# 4585) and frozen in liquid nitrogen. 30-micron cryosections were obtained and stored at −80 °C until stained. Slides were allowed to come to room temperature then placed on a 60 °C slide warmer for 5 min. The OCT mounting compound was dissolved and washed away from tissue sections via 15-min incubation in 1XDPBS supplemented with 0.1% v/v Tween 20 (Fisher, Cat# BP337-500; DPBS-T). Washed tissue sections were then blocked for 15 min at room temperature with 1X DPBS-T supplemented with and 5% w/v bovine serum albumin (RPI Research, Cat# 9048-46-8) in a humidity chamber. Sections were incubated with primary antibodies diluted 1:200 in blocking buffer (Table 3.) overnight at 4C in a humidity chamber. The next day, slides were washed with in 1XDPBS-T and incubated with secondary antibodies diluted 1:500 in blocking buffer overnight, washed, and a coverslip applied with Aquamount (Andwin Scientific, Cat# 41799008) and allowed to dry overnight before imaging.

**TABLE 3:** Vital dyes and antibodies.

Dye or target (hybridoma)	Company	Catalog number	Function
Di-I	ThermoFisher	D282	Vital stain
Nestin (RC2)	Developmental studies hybridoma Bank	RC2	Type VI intermediate filament protein, neural stem cell marker
GFAP	Abcam	ab4674	Astrocyte intermediate filament protein
MBP	Abcam	ab134018	Major protein component of myelin sheath
TuBB3 (Tuj1)	R&D systems	MAB1196	Neuronal class III ß tubulin protein

## 4 Results

### 4.1 Establishing myelination in oligocortical organoids via a xenobiotic-free approach

**FIGURE 2:**
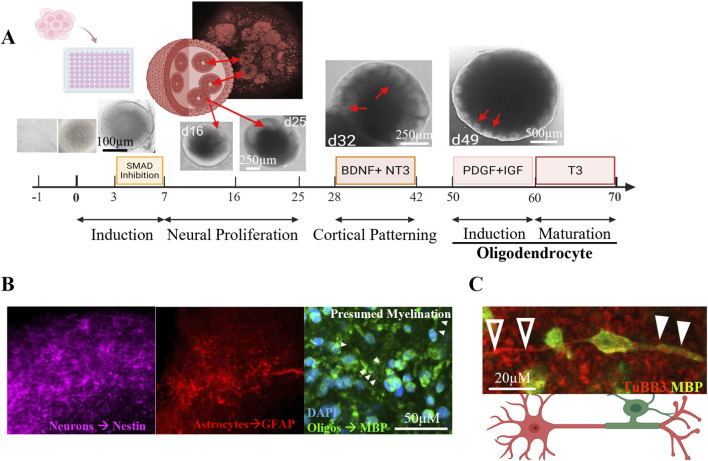
Oligocortical organoids contain neurons, astrocytes, and myelinating oligodendrocytes. **(A)** Human iPSC-derived oligocortical (OC) organoids were differentiated over a 70-day protocol. Neuroepithelial folds (red arrows) emerged around day 14. Cortical patterning was induced at day 28 using BDNF and NT-3. Oligodendrocyte lineage commitment was promoted at day 50 with PDGF and IGF-1, and maturation was enhanced at day 60 with triiodothyronine (T3). **(B)** Immunostaining revealed the presence of Nestin (+) neurons, GFAP (+) astrocytes, and MBP(+) oligodendrocytes within oligocortical organoids. **(C)** MBP(+) oligodendrocytes were observed in close proximity to TuBB3(+) axons (closed arrowheads), suggestive of active myelination.

Following the implementation of our xenobiotic-free protocol to differentiate oligocortical organoids, we consistently generated 96 spheroids per 96-well V-bottom low-attachment plate. To minimize size variability between organoids, we started with a single-cell solution, by CTS-TrypLE dissociation. Additionally, the plates were centrifuged after seeding 10,000 cells per well to promote spheroid formation. By day 3 of differentiation, the spheroids exhibited robust morphological integrity, forming compact and uniform structures (Figure 2A).

Over the course of 70 days, the spheroids progressed through defined stages of neuronal development, guided by the sequential addition of morphogens and growth factors. Neural induction was initiated via dual SMAD inhibition using dorsomorphin and SB-431542. These compounds promote neural specification via inhibition of BMP and TGF-ß signaling pathways (Madhu et al., 2016; Wattanapanitch et al., 2014). Of note, high dorsomorphin concentrations are linked to cytotoxicity, evident by failure to form spheroids by day 3. The dorsomorphin concentration may need to be optimized for each iPSC line used. We recommend seeding a 96 well V-bottom plate with 10,000 cells exposing them to SB-431542 and increasing dorsomorphin concentrations from 1 to 10µM, choosing the highest non-cytotoxic concentration. By day 7, early neural features were evident, including increased cellular density and visible growth.

On day 12, spheroids were transferred to ultra-low attachment plates to facilitate handling and promote further maturation. To support neuronal survival, maturation, and synaptogenesis, BDNF and NT-3 were added at days 28 through 39 (Nieto-Estévez et al., 2022).

Oligodendrocyte specification was induced on day 50 by supplementation with PDGF-AA and IGF-1. PDGF-AA promotes oligodendrocyte precursor cell (OPC) expansion and maturation (Yao et al., 2017), while IGF-1 enhances oligodendrocyte survival and myelination (VonDran et al., 2011). OPC maturation into myelinating oligodendrocytes was further supported by the addition of T3. Although ketoconazole has been described as an alternative inducer of oligodendrocyte maturation (Madhavan et al., 2018), we did not test it in this protocol.

By day 70, the spheroids exhibited hallmark features of cortical neural tissue, including distinct populations of neurons, astrocytes, and oligodendrocytes (Figures 2B,C), consistent with previous reports using xenogenic protocols (Madhavan et al., 2018; Eigenhuis et al., 2023). Neurogenesis was confirmed by Nestin staining, which marked neural progenitors, while TuJ1 (TuBB3) staining identified mature neurons. Astrocytes were visualized via GFAP staining, indicating widespread astrogliogenesis.

Importantly, we observed maturing oligodendrocytes. Myelin basic protein (Snaidero et al.), a critical component of CNS myelin synthesized locally at axon-glial contact sites (Snaidero et al., 2017), was expressed in oligodendrocytes adjacent to TuBB3+ neurons. Expression of MBP in membrane extensions that wrap around axons strongly suggest that the oligodendrocytes present in our organoids have reached a mature, myelinating stage (Müller et al., 2013). The proximity of MBP and TUBB3+ neurons further suggests the presence of functional myelination within the organoids (Figures 2B,C). However, we observed MBP expression near oligodendrocyte somas that became “patchy”, suggesting incomplete myelination and tissue maturation.

These results demonstrate that our xenobiotic-free protocol reliably produces oligocortical organoids containing specified neural cell types and oligodendrocytes, offering a promising platform for modeling cortical development and myelin-related disorders such as MS.

### 4.2 Xenobiotic-free generation of retinal organoids with accurate architecture and migrating RGC axons

To facilitate the transition to clinically relevant culture systems, we developed a simplified xenobiotic-free protocol for differentiating human iPSCs into retinal organoids. To assess the fidelity of retinal development, we utilized a Brn3-GFP reporter iPSC line to aid in RGC visualization during specification and maturation.

**FIGURE 3:**
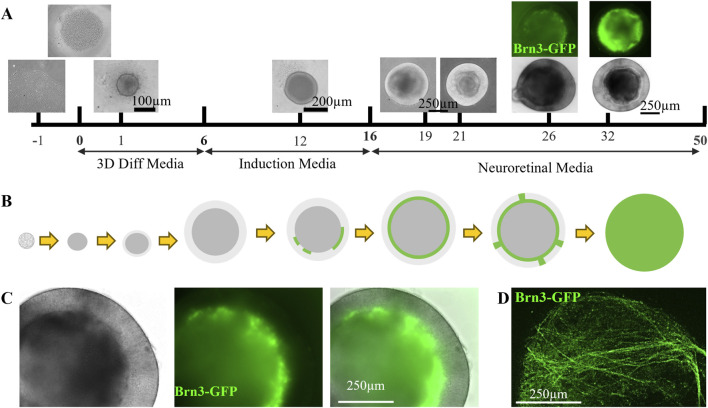
Retinal organoids containing RGCs recapitulate tissue architecture and function. **(A,B)** iPSCs were dissociated into single cells and plated in ultra-low attachment plates with 3D differentiation media. Retinal fate was induced at day 6 using cytokine-enriched media for 10 days. Using a Brn3-GFP reporter line, GFP(+) retinal ganglion cells (RGCs) were detected by day 21 and fully populated the inner neuroretinal surface by day 32. Organoids were matured through day 50, at which point RGC axon projection intensity precluded individual cell visualization via epifluorescence. Protocol adapted from Harkin et al. (2024) and Wiley et al. (2017). **(C)** High-magnification imaging of day 30 retinal organoids confirmed appropriate Brn3-GFP(+) RGC localization. **(D)** Confocal microscopy revealed RGC axons projecting and encircling the organoid surface, suggestive of active pathfinding.

To reduce inter- and intra-cohort variability, we began with a single-cell suspension. Healthy iPSC colonies were dissociated using CTS-TrypLE and seeded at a density of 4,000 cells per well in 3D retinal differentiation media into 96-well V-bottom ultra-low attachment plates (Figure 3A). Centrifugation was used to concentrate the cells at the bottom of each well, promoting uniform spheroid formation.

Retinal fate was induced by the addition of BMP4 on day 6, following a modified protocol adapted from Harkin et al. (2024) and Wiley et al. (2017) (Harkin et al., 2024; Wiley et al., 2017). By day 21, GFP + RGCs began to emerge and progressively populated the inner neuroretinal surface. By day 32, Brn3-GFP + cells formed a dense layer along the inner face of the organoid (Figure 3A), consistent with the spatial organization of the ganglion cell layer *in vivo* (Li et al., 2021; Mellough et al., 2019).

Retinal organoids were matured through day 50, at which point RGC axons extended around the organoid exterior. Due to this extensive axonal outgrowth, individual GFP + cells became difficult to resolve via epifluorescence imaging (Figure 3B). Confocal microscopy at day 30 confirmed appropriate localization of Brn3-GFP + cells within the inner retinal layer (Figure 3C) and revealed axonal projections encircling the organoid surface (Figure 3D). This behavior is consistent with axonal pathfinding and potential synaptic targeting, as previously described (Drabbe et al., 2025).

Altogether, these findings confirm that our xenobiotic-free protocol reliably generates retinal organoids containing spatially organized RGCs. To better mimic the *in vivo* environment, we generated assembloids by fusing retinal organoids with brain-derived structures following completion of the differentiation protocols.

### 4.3 Modelling retinocortical connectivity in xenobiotic-free assembloids

**FIGURE 4:**
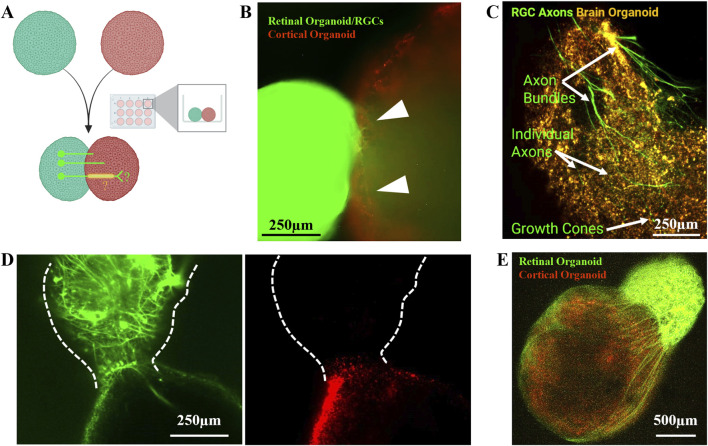
3D assemblies of retinal and oligocortical organoids demonstrate unidirectional axonal migration. **(A)** Mature Brn3-GFP(+) retinal organoids (green) and DiI-labeled oligocortical organoids (Nieto-Estévez et al.) were co-cultured in slanted 12-well plates and left undisturbed for 3 days. **(B)** By day 3 post-assembly, RGC axons (green, arrowheads) began migrating onto and into the oligocortical organoid surface. **(C)** By day 7, axons had reached the opposite pole of ∼2 mm oligocortical organoids, migrating at ∼300 µm/day. Axons travelled individually and in bundles, both on the surface and through the parenchyma, and exhibited growth cones. **(D)** Migration was unidirectional, originating from the retinal organoid and targeting the oligocortical organoid. **(E)** Whole-mount immunofluorescence of ethyl cinnamate-cleared assembloids revealed dense RGC axon bundles.

To investigate the connectivity between the retina and the brain differentiated with our xenobiotic-free protocols, we assembled mature Brn3-GFP + retinal organoids with Di-I (ThermoFisher, Cat# D282) labelled oligocortical organoids. Di-I labelling, was performed to follow oligocortical organoid derived cells. To generated assembloids, individual organoids were placed in contact with each other in a low attachment 12-well plates (Figure 4A), tilted to a ∼45° angle, and left undisturbed for 3 days.

We assessed RGC axon migration across tissue boundaries, building on previous assembloid models (Miura et al., 2022; Fligor et al., 2021). By day 3 post-assembly, Brn3-GFP + RGC axons were observed migrating onto the surface and into the parenchyma of the oligocortical organoid (Figure 4B). Initially axons extended individually but were also observed in bundles by day 7. Bulges observed at the distal tip of migrating axons are consistent with growth cones, indicating the potential to actively observe axonal pathfinding in living human tissue, as described by others (Fligor et al., 2020; Sridhar et al., 2020).

By day 7, retinal ganglion cell axons had traversed the entire ∼2 mm diameter of the cortical organoid, migrating at an estimated rate of ∼300 µm/day (2 mm/7days = 285.7 µm/day; Figure 4C). Migration occurred both superficially and through the organoid parenchyma. Notably, axonal migration was unidirectional, originating exclusively from the retinal organoid and targeting the oligocortical organoid (Figure 4D), suggesting intrinsic directional cues and potential synaptic targeting. Live imaging of assembloids was optimized via iodixanol refractive index matching and stable for at least 48-day of co-culture. Live imaging revealed dense RGC axon bundles encircling and penetrating the cortical organoid (Figure 4F).

These results demonstrate that retinal-cortical assembloids support stable, directed axonal migration, recapitulating aspects of the development and differentiation of RGCs *in vivo*. In our hands, assembloid formation was dependent on the organoids making contact during the initial 3-day incubation period. It was crucial to ensure that the tilted co-cultures were not disturbed during their initial assembly. We found it best to ensure organoids were still touching once transferred to the incubator from the biosafety cabinet, to avoid disturbing co-cultures by gently open/closing incubator doors, and to place the tilted plate(s) at an easily accessible level within the incubator (not the top of stacked incubators) to visually inspect each co-culture. Altogether the protocols established here present a novel framework for modeling RGC development and maturation in a relevant, xenobiotic-free microenvironment–enabling not only the study developmental processes but also the investigation of complex diseases.

## 5 Discussion

### 5.1 Assembloids faithfully mimic the retinofugal projection

*In vitro* modeling of complex disease processes involving the visual pathway using organoids necessitates development of more complex models that more closely recapitulate the *in vivo* visual system. Current *in vitro* models of optic neuropathy are insufficient; highlighting a critical need for the development of more accurate human organotypic and microphysiologic systems (Strat and Ganapathy, 2025). The methodology described here aims to address this need and provides a foundation upon which to build.

We generated xeno-free methods for growing oligodendrocytes within cortical tissue, as well as xeno-free retinofugal assembloids. Notably, we introduce a protocol to produce retinofugal assembloids that incorporate oligodendrocytes—a crucial step towards the generation of accurate organotypic models of the anterior visual pathway. The described xeno-free approach enhances the physiological relevance of the system by eliminating animal-derived components, thereby supporting a fully human microenvironment. Such models offer valuable insights into human-specific developmental processes, disease mechanisms, and preclinical evaluation of cellular and pharmaceutical therapies. However, additional characterization of the organoids and assembloids generated by the described methods will be essential to widespread adoption.

Among current strategies, GMP-compliant retinal organoids derived from human iPSCs stand out for their fully defined, xeno-free media and controlled oxygen conditions. This protocol enables production of transplantable photoreceptor precursors that survive and integrate *in vivo* (Bohrer et al., 2025), In parallel, xeno-free cortical spheroids generated under feeder-free conditions demonstrated long-term viability and functional neuronal networks (Sloan et al., 2018; Miura et al., 2020). Finally, recent work integrating retinal organoids with microfluidic chip system platform offers a scalable approach to mimic human physiology, in a xeno-free environment (Chen et al., 2017). In contrast, our approach prioritizes simplified culture conditions and reduced technical complexity in a xeno-free environment, which facilitates broader accessibility and reproducibility, even though it lacks perfusion dynamic offered by the chip-based systems. Building on this foundation, our assembloid system successfully recapitulates key aspects of RGC development, differentiation and maturation. Furthermore, the observed axonal integration between the retinal and the oligocortical organoids underscores the potential of this platform to more closely model the anterior visual pathway that closely mirrors human physiology, providing a robust platform for future investigations into neurodevelopment and pathogenesis.

**FIGURE 5:**
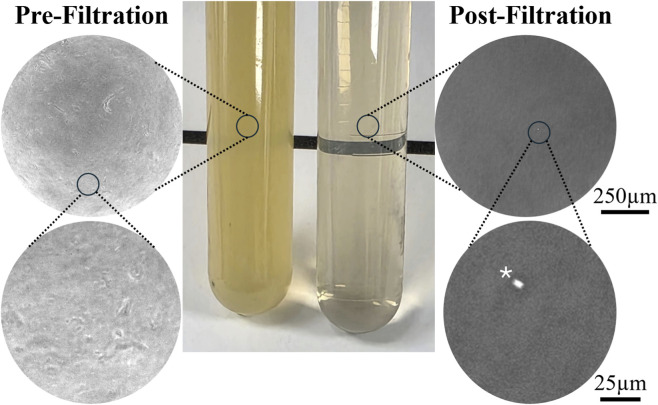
Removal of insoluble material from pooled human serum is essential freshly thawed heat inactivated human serum is a turbid yellow solution (center left) with myriad insoluble particles (far left micrographs). Sequential filtration through 0.80 µm, 0.45 µm, and 0.22 µm PES membranes effectively removes insoluble particles (far-right micrographs) with markedly reduced turbidity and transition to a pale straw color (middle left versus middle right). Note, asterisk (*) indicates debris on the bottom of the plate that was used as a focal point. It is not from the serum filtrate.

Building on the physiological relevance of our xeno-free assembloid system, we refined the culture conditions by pairing human serum with a defined recombinant human extracellular matrix cocktail. This strategy eliminates the variability and confounding factors associated with animal-factor containing reagents, facilitating further refinement for use in cell-therapy protocols. While the heat inactivated human serum offers a xeno-free alternative, our experience highlights the challenges associated with its use. We have observed lot-to-lot variability, and in some extreme instances, we noted variability between preparations from the same lot when culturing primary cells (data not shown). This variability included altered growth rate, which has been reported by other groups attempting to develop xeno-free GMP compliant retinal organoid differentiation protocols (Hays et al., 2025). We noticed that insoluble particles—likely aggregated serum proteins resulting from the heat inactivation process—appeared to contribute to the observed development issues (Soltis et al., 1979). However, the removal of these particles proved time-consuming and technically challenging. Our preferred approach is stepwise filtration of thawed and warmed human serum, sequentially passing it through 0.8micron, 0.45micron, and 0.22micron filters. As seen in Figure 5, this filtration system effectively removed insoluble material and associated growth delay. Gross visual confirmation of successful filtration—loss of turbidity—with microscopic confirmation (e.g., place 100 µL of filtered serum into 96-well plate and observe under phase contrast) has been sufficient. This filtration-based approach has minimized variation and was instrumental in establishing the xeno-free protocol. Future studies will aim to further characterize the serum properties that support differentiation and provide the field with guidelines (e.g., turbidity, protein and glucose concentrations) to increase reproducibility and consistency across cell lines and laboratories. Additionally, the use of V-bottom plates greatly improved our ability to generate a single, round spheroid per well. The slight variability observed in spheroid formation is likely due to insufficient resuspension or settling of cells resulting in pipetting errors or insufficient dissociation (e.g., cell clumps leading to asymmetry). During the retinal organoid differentiation, we noticed that half-media changes in a 96-well plate with a small 4000-cell spheroid are labor-intensive and, at times, difficult. Thus, we have made significant efforts to limit the number and frequency of media changes, especially at highly vulnerable early time-points. Moreover, our method includes thrice-weekly media changes which also provides opportunity to implement this workflow into weekly schedules, giving scientists respite over the weekend.

## 6 Implication of findings

The protocols described here are approachable and translatable to clinically compatible methods. Funding agencies and regulatory bodies are increasingly supportive of “New Approach Methods” (NAMs). Compared to traditional animal model heavy preclinical testing, NAMs rely on *in silico* and/or *in vitro* models based on human cells and/or tissue—such as the organoids and assembloids described here. The methods described do not require specialized equipment nor a technologically advanced skillset to master. Furthermore, we focused on the use of readily available reagents, culture dishes, and basic methodology such that it is compatible for wide adoption. Additionally, the standardization of cell numbers and avoidance of adherent steps facilitates generation of uniformly sized organoids.

The impact of the described novel xeno-free method to generate visual system assembloids extends well beyond MS-related ON. These protocols are readily amenable to development of glaucoma-related optic neuropathy models, MOGAD, NMOSD, and other forms of ON. These methods can be utilized for investigation of the underlying pathomechanisms driving genetic and acquired forms of optic neuropathies and autoimmune mediated ON (Cheng and Kuehn, 2023). Retinofugal assembloids can be used to build upon this work for evaluation of therapeutic drug toxicities as well as neurotoxic environmental exposures (such as burn pit smoke, wildfires, air pollution, etc.). Furthermore, this xeno-free protocol bypasses the adherent step and need to dissect retinal cups from non-retinal tissues seen in other protocols, making the protocol described here scalable through adaptation to automated platforms and further facilitate translation. This includes development of preclinical high-throughput platforms for pharmaceutical development that rely on human tissue rather than animal models. Additionally, the xeno-free nature of our approach is a significant step towards Good Manufacturing Practice (GMP) compatibility and development of cell-based therapies. This includes development of oligodendrocyte replacement for treatment of demyelinating disorders and implicates assembloids as a superior source of RGCs for cell-replacement strategies to treat MS-related ON, glaucoma, and related diseases typified by RGC loss (Soucy et al., 2023; Johnson et al., 2023).

## 7 Study limitations and future directions

The protocols presented here provide a simple and accessible method to generate xeno-free organoids and assembloids that mimic the structural organization of the anterior visual pathway. We include qualitative assessment and expected outcomes to guide users but urge caution when interpreting these results. For instance, the current study lacks quantitative assessment of the proportion of differentiated vs. undifferentiated cells within individual organoids. We demonstrate the described culture conditions that support expression of markers associated with mature oligodendrocytes (MBP) and neurons (TUBB3) suggesting the maturation of cells. However, this assessment does not provide definitive evidence of maturity or functionality of these cell types.

We rely on a genetically encoded Brn3-GFP reporter to demonstrate the presence of RGCs and projection of their axons. This analysis is limited and does not provide insight into all RGC sub-types, other retinal cell types, nor the presence of undifferentiated cells. Our reliance on Brn3-GFP does not allow us to differentiate GFP-expression by RGCs to that of non-target cells. Retinal expression of Brn3 is limited to RGCs, yet multiple CNS neurons are known to express Brn3—our approach does not facilitate differentiation between RGCs and aberrant differentiation of Brn3+ CNS tissue within retinal organoids. This strategy further limits our ability to demonstrate RGC functionality, maturity, and connectivity. Future studies aim to expand on the presented results and close these gaps.

With our xeno-free assembloid model we created an *in vitro* microenvironment capable of supporting RGC maturation. These oligodendrocyte rich assembloids have the capacity to myelinate cortical as well as RGC axons. We are currently pursuing studies to further understand this phenomenon, as well as further characterization of the reproducibility, such as incorporation of additional iPSC lines and maturity of the tissues produced by these methods. To this end, we have also begun to characterize and define the properties of filtered human serum that support organoid differentiation, with the goal of providing quality control guidelines.

Assembly of iPSC derived organoids into increasingly complex tissues brings the promise of increased functionality. Future studies should focus on investigating the functionality of retinal, oligocortical, and retinofugal assembloids generated using the described methods. Our current studies include investigation of synapse formation and electrical activity in response to light stimuli. However promising, the results of these experiments are premature and beyond the scope of the presented work. Similarly, the presented results demonstrate oligodendrocyte MBP expression including process extension and association with neurons suggesting active myelination. However, further analysis, including ultrastructural and electrophysiologic measurements, are required to definitively demonstrate the presence of myelination in these cultures, as well as functional measurements including axonal migration, pathfinding, and synapse formation.

These future studies include incorporation of thalamic rather than cortical tissue and incorporation of microglia, which likely influence RGC synapse formation and myelination. To this end, we hypothesize that generation of a complete and functional anterior visual pathway—from photoreceptor to thalamic interneuron—requires the correct brain region (thalamic organoid) and full complement of glial cells (microglia, astrocytes, and oligodendrocytes). Thus, we will build upon the described methods to generate a cellularly complete and anatomically accurate *in vitro* model of the human visual pathway through assembly of iPSC derived retinal, thalamic, and cortical organoids, including oligodendrocytes and iPSC derived microglia. Here, single-cell transcriptomic analysis will be used to compare organoids to assembloids and provide quantitative measures of the proportion of differentiated versus undifferentiated cells, catalog the cell-types with relative distribution, and expression profile. This transcriptomic analysis will be paired with histopathological, functional, and ultrastructural analysis to fully characterize the tissue generated by these methods.

Building on this comprehensive characterization, xeno-free assembloid based models are likely to provide superior platforms for disease modeling. In addition to their physiological relevance, assembloids are likely to be more amenable to *in vitro* assays of retinal electrical activity and connectivity. This could be accomplished through incorporation of genetically encoded calcium indicators in RGCs and/or their brain region targets or directly though patch clamp or electrode array (Soltis et al., 1979). For instance, the adaptive immune system contributes to the etiology and progression of glaucoma and autoimmune demyelinating disorders, and these immune-related mechanisms can be easily influenced by the incorporation of animal products (Shestopalov et al., 2021; Attfield et al., 2022). Thus, we will build upon the successful generation of retinofugal assembloids by transitioning to disease modeling. This includes incorporation of cytokine mediated inflammatory injury, patient derived immunoglobulin, patient derived cells, and demyelinating injury (Gramlich et al., 2022; Owens et al., 2023; Gramlich et al., 2020). The methods described within this manuscript are foundational to the development of clinically applicable therapies for treatment of glaucoma, MS, and other diseases that manifest in the form of optic neuropathy.

## Data Availability

The original contributions presented in the study are included in the article/supplementary material, further inquiries can be directed to the corresponding author.
